# Macular Ganglion Cell–Inner Plexiform Layer as a Marker of Cognitive and Sensory Function in Midlife

**DOI:** 10.1093/geroni/igaa057.832

**Published:** 2020-12-16

**Authors:** Natascha Merten, Adam Paulsen, A Alex Pinto, Yanjun Chen, Lauren Dillard, Mary Fischer, Carla Schubert, Karen Cruickshanks

**Affiliations:** University of Wisconsin-Madison, Madison, Wisconsin, United States

## Abstract

Neurodegenerative diseases are public health challenges in aging populations. Early identification of people at risk for neurodegeneration might improve future treatment. Noninvasive, inexpensive screening tools are lacking but of great potential. Optical coherence tomography (OCT) measures nerve cell layer thicknesses in the retina, which is an anatomical extension of the brain and might be reflective of generalized neurodegeneration. We aimed to determine associations of macular ganglion cell-inner plexiform layer (mGCIPL) thickness with cognitive and sensorineural function in midlife. This study included 1880 Beaver Dam Offspring Study participants from the 10-year follow-up examination. We assessed cognition (principal component analysis of multiple cognitive test scores), cognitive impairment, hearing sensitivity thresholds and impairment, central auditory processing (% correct on a dichotic digits test), and visual and olfactory impairment. We measured mGCIPL using the Cirrus 5000 HD-OCT Macular Cube Scan. Multivariable linear and logistic regression models were used to determine associations of mGCIPL thickness and thin mGCIPL, defined as 1 standard deviation below average, with cognitive and sensorineural functions. Thinner mGCIPL was associated with worse cognition (0.01 standard deviation increase per µm thickness;95% confidence interval (CI) 0.01,0.02;p<.0001), worse central auditory function (0.07% increase per µm thickness;CI 0.01,0.13;p=.03) and visual impairment (Odds Ratio=0.95;CI 0.94,0.97;p<.0001). MGCIPL thickness was associated with hearing sensitivity in women only. There were no associations with impairments in hearing, olfaction and cognition. Results for thin group comparisons were consistent. MGCIPL thickness is associated with cognitive and sensorineural function and has potential as a marker for neurodegeneration in middle-aged adults.

